# *EsigGOBP1*: The Key Protein Binding Alpha-Phellandrene in *Endoclita signifer* Larvae

**DOI:** 10.3390/ijms23169269

**Published:** 2022-08-17

**Authors:** Ping Hu, Enhua Hao, Zhende Yang, Zhisong Qiu, Hengfei Fu, Jintao Lu, Ziting He, Yingqi Huang

**Affiliations:** 1 Forestry College, Guangxi University, Nanning 540003, China; 2 Forestry College, Beijing Forestry University, Beijing 100083, China

**Keywords:** general odorant-binding proteins, eucalyptus, molecular docking, qRT-PCR

## Abstract

*Endoclita signifer* larvae show olfactory recognition towards volatiles of eucalyptus trunks and humus soils. Further, *EsigGOBP1* was identified through larval head transcriptome and speculated as the main odorant-binding proteins in *E. signifer* larvae. In this study, the highest expression of *EsigGOBP1* was only expressed in the heads of 3rd instar larvae of *E. signifer*, compared with the thorax and abdomen; this was consistent with the phenomenon of habitat transfer of 3rd instar larvae, indicating that *EsigGOBP1* was a key OBP gene in *E. signifer* larvae. Results of fluorescence competition binding assays (FCBA) showed that *EsigGOBP1* had high binding affinities to eight GC-EAD active ligands. Furthermore, screening of key active odorants for *EsigGOBP1* and molecular docking analysis, indicated that *EsigGOBP1* showed high binding activity to alpha-phellandrene in 3rd instar larvae of *E. signifer*. Conformational analysis of the *EsigGOBP1*-alpha-phellandrene complex, showed that MET49 and GLU38 were the key sites involved in binding. These results demonstrated that *EsigGOBP1* is a key odorant-binding protein in *E. signifer* larvae, which recognizes and transports eight key volatiles from eucalyptus trunk, especially the main eucalyptus trunks volatile, alpha-phellandrene. Taken together, our results showed that *EsigGOBP1* is involved in host selection of *E. signifer* larvae, which would aid in developing *EsigGOBP1* as molecular targets for controlling pests at the larval stage.

## 1. Introduction

Insects have evolved an extremely sensitive olfactory system, involved in distinguishing trace odor components from the ambient complex environment, to exhibit corresponding behavioral responses [1]. The olfactory system of insects plays an important role in feeding, mating, oviposition and avoidance of natural enemies or adverse environments [1,2]; it is made up of seven types of olfactory proteins which includes odorant-binding proteins (OBPs), chemosensory proteins (CSPs), sensory neuron membrane proteins (SNMPs), odorant receptors (ORs), gustatory receptors (GRs), ionotropic receptors (IRs), and odor-degrading enzymes (ODEs) [3]. Importantly, the initial step of odorant recognition is suggested to involve the transport of odor molecules by the OBPs across the aqueous sensillum lymph to stimulate the ORs, which then activate the olfactory signal transduction pathway [4]. As the first gate involved in odorant recognition, OBPs may serve as molecular targets for the control of moths or other insect species [5,6], and among the large number of OBPs in one species, screened and demonstrated the key OBP involving in main odor recognition was the base of development molecular targets.

OBP are grouped into several classes (Classic OBPs, Dimer OBPs, Plus-C OBPs, Minus-C OBPs, and Atypical OBPs) based on the similarity of their sequences and cysteine number [7,8,9]. Classic OBPs are further grouped into three clusters: pheromone binding proteins (PBPs), general odorant-binding proteins (GOBPs) and antennal-binding proteins (ABPs) based on the similarity of their sequences [10]. The PBPs preferably bind sex pheromones [1], while GOBPs are expressed in the antennae of both sex in Lepidoptera [11]. Further, phylogenetic analysis has found that the sequence identity of lepidopteran GOBPs/PBPs are highly conserved, forming a unique branch in the OBPs family; this suggested that the GOBPs/PBPs evolved from a common ancestor by gene duplication [12,13]. The GOBPs are highly expressed in female and male antennae than in other tissues [10,14]. For example, PsauGOBP1 was found to have significantly lower expression in the proboscises, tarsi, wings, pheromone glands, and hair brushes of *Peridroma saucia* than in antennae [15]. Besides in different developmental stage, OBPs show high expressions at both the larval and adult stages of insects [16]. For examples, at different developmental stage, majority of OBPs in *D. melanogaster* showed the same expression patterns in adult and larval tissues [17]; furthermore, *AglyOBPs* (except for *AglyOBP6*, *8*, and *11*) showed the highest expression in the 4th instar nymphs of *Aphis glycines* [18], which was similar to that reported from studies on *Sitobion avenae* and *Megoura viciae* [19,20]; *in Liriomyza trifolii,* LtriOBP5 showed a high expression at the larval stage [21].

Results from many studies have shown that GOBPs are functionally divergent, binding host plant volatiles, sex pheromone or both. For example, GOBPs displayed strong binding affinities to host plant volatiles in some insect species [12,22]. The fluorescence competitive binding assay (FCBA) result showed that CpunGOBP2 of *Conogethes punctiferalis* displayed the widest binding spectra and correlation with 3-carene and pentyl acetate [23]; EoblGOBP2 of *Ectropis obliqua* was reported to have shown a strong binding affinity to some carbonyl-containing volatiles from tea leaves [24]; DabiGOBP2 of *Dioryctria abietella* was reported to have exhibited ligand specificity to terpenes, especially myrcene, suggesting that these host volatiles might be attractants or deterrents [25]. The GOBP1 from *P. saucia* was reported to have displayed high binding affinities to the behavioral attractant, (Z)-3-hexenyl acetate [15]. Besides GOBPs can also bind sex pheromones as strongly as PBPs, such as in *Spodoptera litura* and *Chilo suppressalis* [26,27]; furthermore in *C. punctiferalis,* an in vivo experiment provided evidence of the involvement of GOBP2 in the perception of sex pheromones [28]. Similarity *BmorGOBP2* of *Bombyx mori* was reported to bind to the sex pheromone (10E,12Z)-hexadecadien-1-ol (bombykol) [29]. The participation of *GOBP2* in the perception of a sex pheromone substance in both larval and adult *S. litura* was unraveled using CRISPR/ Cas9 mutagenesis [30]. Additionally, a few studies have reported that some *GOBPs* showed high binding affinities for both host plant volatiles and sex pheromone components [17,31]. For example, CsupGOBP2 of *C. suppressalis* exhibited not only high binding affinities to five volatiles, but also showed strong binding affinities to all three sex pheromone components [27]. Notably, the sex pheromones of *Plutella xylostella* also work on attracting early-instar larvae to food sources, and they work only when the plant odorants coexist [32], which indicates an association between pheromone attraction and food source. GOBPs have been reported to show high expressions at the larval stage, where it binds to plant volatiles [33]; however, whether the GOBP in *E. signifer* larvae also bind to plant volatiles, to direct them to food sources is unknown.

The ghost moth, *E. signifer* Walker (Lepidoptera, Hepialidae) is a major native polyphagous wood-boring pest of eucalyptus in China, which has caused high economic and ecological losses since it was found in 2007 [34]. The instar larvae hatch and survive in the soil, then translocate to standing trees at the 3rd instar stage, where they bore into stems and damage eucalyptus trunks. Interestingly, even in mixed forest, the 3rd instar larvae can select and damage eucalyptus accurately. A study of the volatile profiles of eucalyptus trunks and humus soils under eucalyptus plantation, combined with results from a Y-tube olfactometer bioassay and GC-EAD, showed that o-cymene was the compound attracting the 3rd instar larvae of *E. signifer*. Additionally, alpha-phellandrene, alpha-pinene, 4-ethylacetophenone, d-limonene, 2-phenyl-2-propanol, and n-butyl ether were identified as candidate attractants [35]; however, a further exploration of the odor adaptation of 5th instar larvae of *E. signifer*, showed that β-pinene was the main attractant at this larval stage, indicating an olfactory plasticity within *E. signifer* larvae [36]. Additionally, transcriptomic analyses of the head and tegument of *E. signifer* larvae, identified 39 olfactory proteins, in which *EsigGOBP1* showed high expression in the head [37] which can be selected as a functional protein target. Therefore, in this study, based on the screened key volatile, we further explored the olfactory functions of *EsigGOBP1* in sensing odors in *E. signifer* larvae by real-time fluorescent quantitative PCR, fluorescence competition binding assays and molecular docking.

## 2. Results

### 2.1. Chemical-Induced Changes to EsigGOBP1 Transcript Expression in E. signifer Larvae

**Figure 1:**
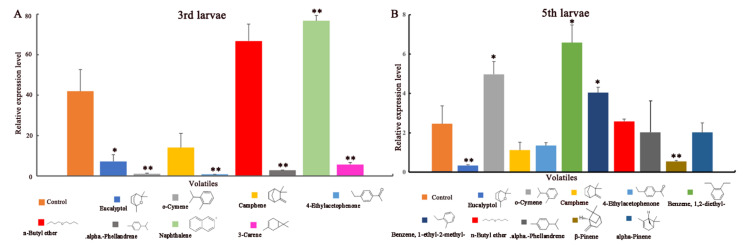
Expression profile of EsigGOBP1 in *E. signifer* larvae from exposure to key volatiles. (**A**) 3rd instar larvae; (**B**) 5th instar larvae. The standard errors are represented by the error bars, * and ** above the bars denote significant differences at *p* < 0.05 and *p* < 0.001 respectively.

Firstly, the expression of *EsigGOBP1* in the head, thorax and abdomen of *E. signifer* larvae were measured. Results showed the *EsigGOBP1* was only expressed in the head of 3rd instar larvae (Figure S1). Secondly, we exposed the 3rd *E. signifer* larvae to eight volatiles (Table S1, 3rd larvae) and then compared the expression of *EsigGOBP1* in the head of treated and control larvae. Tissue-expression profile of the *EsigGOBP1* gene in 3rd instar larvae showed that *EsigGOBP1* expression was significantly lower from the exposure to eucalyptol than the control (*p* < 0.05); extremely significantly lower from the exposure to o-cymene, 4-ethylacetophenone, alpha-phellandrene, and 3-carene than the control (*p* < 0.01); was extremely significantly higher from the exposure to naphthalene the than control (*p* < 0.05). There were no differences in expression between the treatment and control from the exposure to the other volatiles (Figure 1A). In the 5th instar larvae (Table S1, 5th larvae, ten volatiles), exposure to o-cymene, benzene, 1,2-diethyl- and benzene, 1-ethyl-2-methyl- induced significantly higher expression of the *EsigGOBP1* than in the control (*p* < 0.05). Expressions of the *EsigGOBP1* was significantly lower from the exposure to eucalyptol (*p* < 0.05) and extremely significantly lower from the exposure to β-pinene (*p* < 0.01) than the control (Figure 1B).

### 2.2. Coding and Amino Acid Sequences

**Figure 2:**
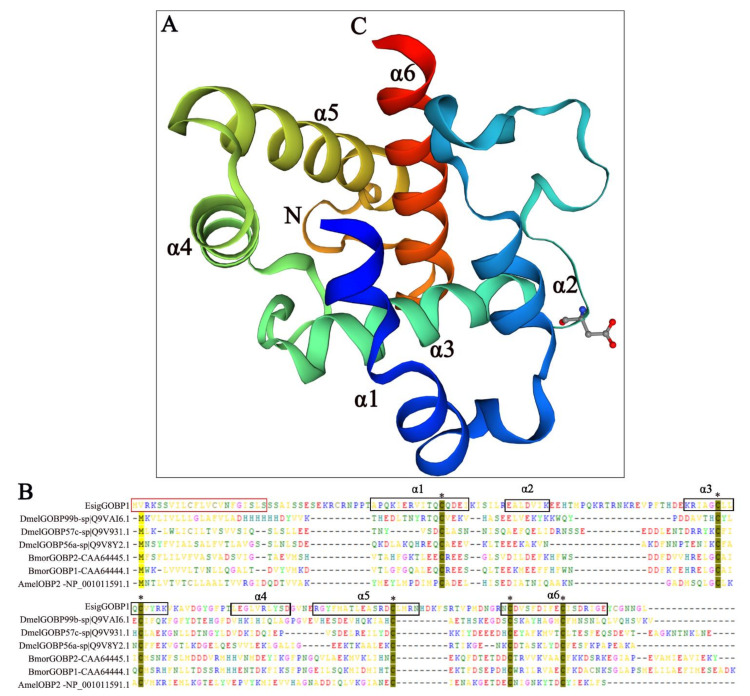
Sequence and structure of *EsigGOBP1.* (**A**) Three-dimensional structure of *EsigGOBP1;* (**B**) Alignment of *EsigGOBP1* with 3D structures of GOBPs in insect and AmelOBP2. The six a-helices with black boxes are as follows, Ala18–Glu23(α1a), Ile26-Ile33 (α1b), Glu40–Lys46 (α2), Lys69–Lys82 (α3), Leu94–Asp103 (α4), Arg108–Asn125 (α5), and Asn140–Glu157 (α6); the red boxes refer to signal peptide; the * refer to six conserved cysteines. The structure was predicted using the SWISS MODEL online tools (http://swissmodel.expasy.org/, accessed on 27 May 2022) and based on *Helicoverpa armigera* pheromone-binding protein PBP1 at pH 7.5 (PDB ID: 7vw8.1.A).

The ORF of *EsigGOBP1* was 561 bp and submitted to GenBank (accession number OP184047), which encoded 187 aa, with a predicted size of 18.76 kDa and an isoelectric point of 7.68, and a 66 aa N-terminal signal peptide. The length of the mature protein is 164 aa. The 3D structure of *EsigGOBP1* was made with the SWISS MODEL (Figure 2A). The QMEAN total score was 0.47, the best identity with a percentage of 11.76%, a similarity of 27% and a coverage of 73% with *Helicoverpa armigera* PBP1 (PDB ID: 7vw8.1.A).The structure contained six α-helices: Ala18–Ile33 (α1), Glu40–Lys46 (α2), Lys69–Lys82 (α3), Leu94–Asp103 (α4), Arg108–Asn125 (α5), and Asn140–Glu157 (α6) (Figure 2B). Six cysteine residues were predicted to form three pairs of disulfide bonds (Figure 2), which corresponded to the known structure of *EsigGOBP1*.

### 2.3. Bacterial Expression and Purification of EsigGOBP1 

*EsigGOBP1* was expressed as an inclusion body, because the recombinant protein was mainly located in the sediment, after lysis and centrifugation of the cells. The *EsigGOBP1* proteins were purified by affinity chromatography, which demonstrated that 40 mM imidazole can be used for protein purification and 200 mM can be used to elute the target protein. In addition, 15% SDS-PAGE was performed and the result showed that there were specific protein bands at approximately 18 kDa (Figure S2). Finally, the protein was denatured and renatured to obtain soluble purified protein. 

### 2.4. Binding Ability of EsigGOBP1 to Host Plant Volatiles

**Figure 3:**
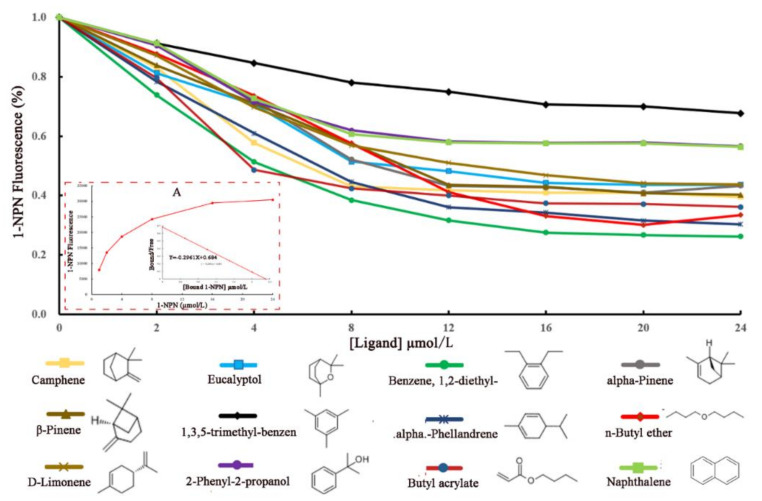
Fluorescence competitive binding assay of EsigGOBP1. (A) The binding curve and Scatchard analysis of EsigGOBP1 and 1-NPN.

In previous studies, results from behavior choice assays to the exposure of eucalyptus volatiles, showed that the 3rd and 5th instar larvae of *E. signifer*, were significantly attracted to only o-cymene and β-Pinene, respectively [33,34]. To determine whether *EsigGOBP1* commonly perceive these GC-EAD active volatiles, we evaluated the binding affinities of recombinant protein *EsigGOBP1* to these volatiles using the fluorescence competition binding assays (FCBA). In accordance with the binding curve and Scatchard plots, *EsigGOBP1* exhibited high affinity toward reporter 1-NPN with a dissociation constant K_1-NPN_ of 3.375 µM (A in Figure 3). The competitive fluorescence binding curves showed that all evaluated ligands reduced the relative fluorescence intensity of the [EhipGOBP1/1-NPN] mixture (Figure 3). In detail, the calculated *K_d_* values ranged from 0.79 µM to 4.60 µM. At the lower level were 0.79 µM for benzene, 1,2-diethyl-, 0.89 µM for alpha-phellandrene, 0.90 µM for butyl acrylate and 0.99 µM for camphene; at the mid-level were 1.18 µM for n-butyl ether, 1.24 µM for alpha-pinene, 1.27 µM for eucalyptol, 1.32 µM for β-pinene and 1.43 µM for d-limonene; at the higher levels were binding to naphthalene, 2-phenyl-2-propanol and 1,3,5-trimethyl-benzendo which recorded *K_d_* were 2.69, 2.77 and 4.60 respectively; however, their concentrations were lower than 50% of the tested concentrations (Figure 3 and Table 1). Thereby the results indicated that EsigGOBP1 was a functional protein allowing *E. signifer* larvae to recognize eucalyptus VOCs, and showed the strongest affinity for benzene, 1,2-diethyl-, alpha-phellandrene, butyl acrylate and camphene. 

### 2.5. Stability and Conformation of EsigGOBP1-Alpha-Phellandrene Complex

**Figure 4:**
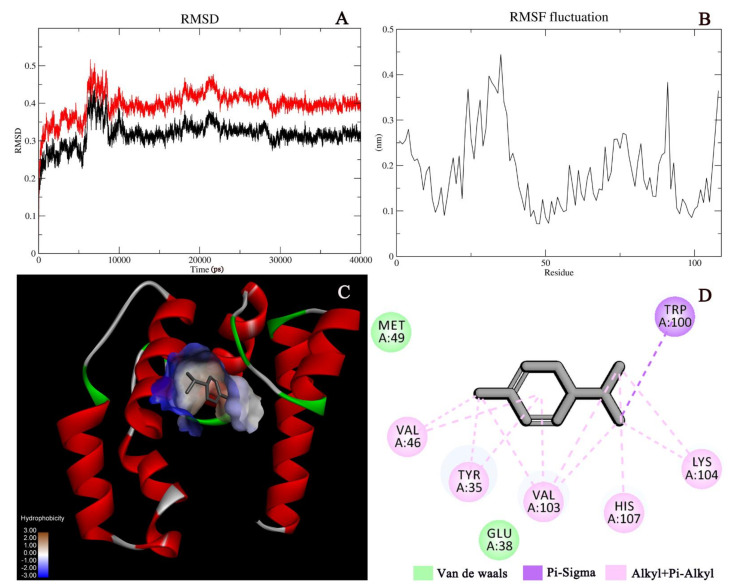
Molecular docking index of the *EsigGOBP1*–alpha-phellandrene complex. The root-mean-square deviation (RMSD) values for carbon backbones of the *EsigGOBP1*–alpha-phellandrene complex during the 40 ns molecular dynamics (MD) simulations (**A**); Residue fluctuations for the *EsigGOBP1*–alpha-phellandrene complex during the 40 ns MD simulations (**B**); Canonical conformations of the *EsigGOBP1-*alpha-phellandrene complex. (**C**) Interactions in the *EsigGOBP1-*alpha-phellandrene complex (**D**).

In this study, the recorded binding energies of EsigGOBP1 to the ligands were between −3.56 kcal/mol and −5.21 kcal/mol (Table 1). Specifically, n-Butyl ether had the highest binding energy of −3.56 kcal/mol, while camphene and alpha-phellandrene had the lowest binding energy of −5.21 and −5.00 kcal/mol, respectively. The binding energy between OBPs and the ligand reflects the strength of the binding between them. The smaller the binding energy is, the more stable the receptor–ligand complex is [38]. Alpha-phellandrene which showed a high binding affinity to EsigGOBP1 in FBCA and a low −5.00 kcal/mol binding energy to EsigGOBP1 was selected for 40 ns molecular dynamics simulations. The MD properties of the EsigGOBP1-alpha-phellandrene complex was analyzed after MD runs through conformation sampling and clusters, and nine clusters were obtained with the top two highest occurrence of 58.10% and 24.00% (Table S2). The RMSD diagram (Figure 4A), showed that a greater part of the protein–ligand system reached a relative equilibrium at around 10 ns. The average RMSD value of the alpha-phellandrene was about 4.0 Å, indicating the stability of the EsigGOBP1-alpha-phellandrene complex. The local motility properties of amino acids residues when the EsigGOBP1 formed a complex with the ligands were further determined by the root-mean-square fluctuation (RMSF) (Figure 4B). Meanwhile, van der Waals, pi-alkyl and alkyl being the most common interaction with alpha-phellandrene; moreover, the existence of GLU38 and MET49, induced an electrostatic force in the complex, which pulled and fastened alpha-phellandrene (Figure 4C,D). In addition, the presence of TYR35, VAL46, TRP100, VAL103, LYS104, and HIS107 provided pi-alkyl and alkyl, and the centroid distance between six key amino acid residue and alpha-phellandrene showed in Figure S3, which also promoted the stability of the binding cavity (Figure 4C,D). 

## 3. Discussion

In this study we evaluated the expressions of *EsigGOBP1* in heads, thorax and abdomen of *E. signifier* larvae, which results showed that it was highly expressed in the heads of 3rd and 5th instar larvae. The FCBA result showed that EsigGOBP1 showed high active binding affinity to eight GC-EAD active ligands. Further, volatile-induced changes in the expressions of the *EsigGOBP1* transcript in the head of 3rd instar larvae, and FCBA and molecular docking results indicated that EsigGOBP1 consistently binds to alpha-phellandrene in the 3rd instar larvae of *E. signifer*; this was further supported by the identification of two key binding sites in the *EsigGOBP1*-alpha-phellandrene complex.

OBPs and GOBPs showed the highest expressions in the head of 5th *E. signifier* larvae [37]; what is more, the highest expression of *EsigGOBP1* showed only expressed in the head of 3rd instar larvae compared to the thorax and abdomen, which is consistent with the phenomenon of habitat transfer at this stage. We speculate that *EsigGOBP1* is a key OBP which plays an important functional role of in olfactory sensing in the head of *E. signifer* larvae. Due to the wee antennae of *E. signifier* larvae, further studies should be carried out on the expression of *EsigGOBP1* and the larval antennae. Besides, OBPs have broad specificity, wide distribution in non-olfactory organs, and involved in multiple non-conventional functions such as taste, immunity response and humidity detection [17,19]; for instance, *TcOBPC17* showed high expression in the head, fat body, epidermis, and hemolymph [39]. The functions of *EsigGOBP1* in the thorax and abdomen of 5th instar larvae of *E. signifier* should explored further. 

As vital factors for selectively solubilizing and transporting external hydrophobic odorant molecules across the lymph, OBPs are involved in odorant molecule discrimination, which could be utilized for screening probable behavioral active compounds of insects [40]. For example, in *B. minax, BminOBP9* was identified as a unique protein that binds multiple citrus VOCs, which can be used to screen probable odors releasing from citrus hosts [41]; furthermore, the *PsauGOBP1* was identified to be involved in detecting host plant volatiles, especially (Z)-3-hexenyl acetate, which can be developed as a probable attractant for the biological control of the species [15]; similar findings were reported for GOBPs in *Maruca vitrata* [22], *Loxostege sticticalis* [42], and Ocimene and (E)-β-Farnesene to AlepGOBP2, and Ocimene to AlepGOBP1 in *Athetis lepigone* [43]. The FCBA results in our study showed that *EsigGOBP1* had high binding affinities for benzene, 1,2-diethyl-, alpha-phellandrene, butyl acrylate, camphene, n-butyl ether, alpha-pinene, eucalyptol, β-pinene and d-limonene, in order of magnitude, but low binding affinities for 1, 3,5-trimethyl-benzen, naphthalene and 2-Phenyl-2-propanol; these probable active compounds were screened and ordered by their *K_d_* values obtained from the FCBA analysis. 

The expression of chemosensory genes can be regulated by host plants [44]. For example, the expression levels of *DcitOBP3*, *DcitOBP6* and *DcitOBP7* were found to change significantly (upregulated or downregulated) in the heads of adult psyllids upon exposure to test volatiles compared with the control [45]. Similar results were observed in the expressions of *AgrnOBPs* in the antennae when exposed to semiochemicals [45]. Further, exposure to the plant kairomone, (E)-2-hexenol, specifically induced the expressions of several *OBP* genes among the 29 *OBP*s that showed higher expressions in *Holotrichia oblita*, and this was further confirmed by an in vitro binding assay [46]. These results suggest that changes in the expression of OBPs may result in the improvement of the detection of semiochemical stimuli. Similarly, we also measured the expression profiles of *EsigGOBP1,* when induced by the exposure to GC-EAD-active volatiles. In detail, the expression of *EsigGOBP1* was upregulated, when the 3rd instar larvae were exposed to naphthalene, but was downregulated when they were exposed to alpha-phellandrene, eucalyptol, o-cymene, 3-carene, and 4-ethylacetophenone; furthermore, the expression of *EsigGOBP1* was downregulated when 5th instar larvae were exposed to β-Pinene and eucalyptol, but was upregulated when exposed to o-cymene, benzene, 1,2-diethyl- and benzene, 1-ethyl-2-methyl-. We speculated that the upregulation and downregulation of *EsigGOBP1* in the 3rd and 5th instar larvae were induced by the detection of these different compounds. On the other hand, the identification of key odorants, which induce the expression of olfactory genes can be used to screen for potential olfactory genes induced by odorants in insects [47]; however, how the upregulation or downregulation of olfactory genes are influenced by time or insect status is unknown. In addition, the mechanism underlying changes in the expression of OBP genes, as induced by odorants remains unclear and requires further research.

FCBA result showed that GOBP with broad spectrum, such as CsasGOBP1–2 displayed high binding affinity to general components of plant volatiles [31] and CpunGOBP2 displaying the widest binding spectra [23]. Furthermore, ApisOBP9 shows high and specific affinity to linear alcohols and aldehydes of 16-carbon [48] and EoblOBP6 binds the tea plant volatile, benzaldehyde and display high binding ability to herbivore-induced plant volatiles (HIPVs), nerolidol and α-farnesene [49] with the narrow spectrum. Similarity, in our previous studies, FCBA result showed that *EsigGOBP1* mainly bind benzene, 1,2-diethyl-, alpha-phellandrene, butyl acrylate and camphene. Interestingly the FCBA result and the expression profile of *EsigGOBP1* from the exposure to GC-EAD active compounds, together showed that alpha-phellandrene was a major volatile from eucalyptus trunk which showed GC-EAD activity in 3rd and 5th instar larvae of *E. signifer* [35,36]; it was also the volatile which showed the second highest binding affinity to *EsigGOBP1,* and only caused down regulation of the gene in 3rd instar larvae. These indicated that alpha-phellandrene was the key ligand of *EsigGOBP1*, as they exhibited an efficient and reactive binding in 3rd instar larvae. In addition, molecular docking analysis showed that alpha-phellandrene had the second lowest binding energy, in its binding to *EsigGOBP1*, which was a further confirmation that it was the key ligand of *EsigGOBP1,* and demonstrated the importance of *EsigGOBP1* in *E. signifier* larvae. Similarly, *DabiGOBP2* [25] and *CsupGOBP2* [27] were reported to have shown the highest binding affinity to myrcene, AlepGOBP1 only bind ocimene in *A. lepigone* [43], and SzeaOBP28 exhibited a pronounced binding affinity for 4-hydroxy-3-methoxybenzaldehyde in behavioral experiment and by FCBA [50]. 

The binding affinities of OBPs for ligands are usually determined using fluorescence competitive binding assays using recombinant proteins [51]; however, in vitro binding assays may produce false positive results because candidate ligands with high binding affinities may exhibit weak effects on behavioral responses [52]. In our study, we observed that benzene, 1,2-diethyl- and alpha-phellandrene, had no obvious influence on the behaviour of *E. signifier* [35,36], and may be the reason for the difference between the FBCA and binding energy values. Besides in molecular docking analysis, binding energies are used to evaluate the strength of the protein’s binding ability to the ligand. The binding energy is negatively correlated with the binding ability, with a lower binding energy indicating a more spontaneous reaction and a higher binding capacity and vice versa [51]. The order of alpha-phellandrene, alpha-pinene and eucalyptol in terms of their binding with *EsigGOBP*, showed the same consistency between binding energy and FCBA results, but others did not show such consistency. 

Although the amino acid sequences of insect OBPs are highly divergent between and within species, the structures of insect OBPs are highly conserved. The three-dimensional structure of classic OBPs consists of a six-helical domain forming a hydrophobic cavity [53]. The structural stability of insect OBPs depends on the presence of three interlocked disulphide bridges linking conserved cysteines [54,55]. The binding domains of insect OBPs have also been identified in some insect species. For example, six amino acid sites of MvitOBP3 were identified to be involved in the binding of the hostplant volatiles [56]; furthermore, Trp37 was identified as a key site in the *A. polyphemus* PBP and which may be playing an important role in the initial interaction with ligands of, while Asn53 plays a critical role in the specific recognition of pheromones [57,58]. In our study we identified eight amino acid sites in *EsigGOBP1* (GLU38, MET49, TYR35, VAL46, TRP100, VAL103, LYS104, and HIS107) involved in the binding of alpha-phellandrene, with MET49 and GLU38 identified as key sites; however, further studies on the binding and release mechanisms of the *EsigGOBP-*alpha-phellandrene complex are required.

## 4. Materials and Methods

### 4.1. Expression Analysis of Olfactory Proteins after Exposure to Volatiles

The 3rd and 5th instar larvae of *E. signifer* were exposed to 12 GC-EAD active volatile compounds (Table S1). The exposure method followed that of Llopis-Gimenez and Hu [35,59]. Thirty-three 5th instar larvae were exposed to ten compounds and only methanol (control) with three biological replicates (50 μL of each odorant diluted to 10 g/L in methanol in a 50 mL jar for 24 h). Thereafter, the total RNA from the heads of these larvae were extracted and its concentration and purity measured. The residual genomic DNA in the total RNA was removed using DNase I (Thermo Scientific, Waltham, MA, USA) then purified RNA used to synthesize cDNA following the method of Zhang [37]. The treatments carried out on the 5th instar larvae, were the same as applied to 3rd instar larvae (n = 27) (eight compounds, one control; three biological replicates). The sequence of the gene-specific primers of *EsigGOBP1* were as follow: forward primer: 5′-GAAGATCGAGC GGGTGATTA-3′; reverse primer: 5′-TCCAGCAATCCTCT TCTCGT-3′ [37]. Roche LIGHT CYCLE 480II (USA) and Genious 2X SYBR Green Fast qPCR Mix (No ROX) (No. RK21205; ABclonal, Wuhan, China) were used for a PCR reaction under a three-step amplification program. Each 20 µL real-time fluorescence quantification PCR (qRT-PCR) reaction mixture contained 10 µL of Genious 2X SYBR Green Fast qPCR Mix, 0.8 µL of each primer (10 mM), 2 µL of sample cDNA (2.5 ng of RNA), and 7.2 µL of dH_2_O (sterile distilled water), with the following cycling parameters: 95 °C for 180 s, followed by 40 cycles of 95 °C for 5 s, 60 °C for 30 s, and 65 °C to 95 °C in increments of 0.5 °C for 5 s to generate melting curves. The sequences of the primers of the reference gene, GADPH were forward primer 5′-TTGACACCGACGACAAACAT-3′; reverse primer 5′-ATTGCTGTTTTCTCGGAACG-3′ [60]. The measurement of the expression of *EsigGOBP1* in head, thorax and abdomen of 3rd instar larvae followed the protocol of Zhang [37]. Each qRT-PCR reaction for each volatile exposure and tissues, was performed in three biological replicates and three technical replicates in Roche LIGHT CYCLE 480II. The expression of *EsigGOBP1*, from exposure to o-cymene and camphene served as the control for the 3rd and 5th instar larvae, respectively, and GADPH was used to measure the relative gene expressions by the 2^−ΔΔCT^ method. The 2^−ΔCT^ method was used to measure gene expressions in the tissues of 3rd instar larvae with 0.91 amplicons efficiencies [61]. Differences in the expression of olfactory proteins were determined using a one-sample *t*-test. All analyses were implemented in SPSS Statistics 18.0, and values are presented as means ± SE.

### 4.2. Cloning and Sequencing

*EsigGOBP1* was amplified by PCR with the gene-specific primers: forward primer, 5′-AGCAGCGCTATATCATCGG-3′; reverse primer, 5′-TCACAGGCC GTTGTTGCCG-3′ and with the cDNA of 12th larvae head (six) as template. The condition of PCR was 98 °C for 10 s, 55 °C for 50 s, and 72 °C for 5 s, for 34 cycles according to ExTaq DNA polymerase (Takara, Dalian, China). The products were ligated into the pEASY-T Easy Vector (TransGen, Beijing, China) to construct recombinant plasmid, then which were converted into *Escherichia coli* DH5α competent cells and plated onto LB agar medium (1 µL ampicillin: 1 mL LB). Positive clones and the amplified DNA was sequenced (Qingke, Beijing, China).

### 4.3. Sequences and Structural Analysis 

Prediction signal peptide of *EsigGOBP1* was used the SignalP 5.0 Server (https://services.healthtech.dtu.dk/service.php?SignalP-5.0, accessed on 9 March 2022) [62]. Sequence alignment was used Mega5.0 (Sudhir Kumar, Koichiro Tamura, and Masatoshi Nei and The Pennsylvania State University), with the sequences of the 3D structure of *GOBPs* in insect and *AmelOBP2*. Prediction three-dimensional models of EsigGOBP1 were conducted by the SWISS MODEL online tools (http://swissmodel.expasy.org/, accessed on 27 May 2022) [63] with SWISS-MODEL template library (SMTL version 1 June 2022, PDB release 27 May 2022, searched with BLAST) [64]. HHBlits [65] with default parameters was performed for evolutionary related structures matching EsigGOBP1. The *Helicoverpa armigera* pheromone-binding protein, PBP1 at pH 7.5 (PDB ID: 7vw8.1.A) was chosen as an appropriate template for homology modeling of EsigGOBP1. The templates with the highest quality were then selected for model building. Models were built based on the target-template alignment using ProMod3 [66] with default parameters. 

### 4.4. Recombinant Expression and Purification

Restriction enzymes (*Hind*III and *EcoR*I) were used to constructed the prokaryotic expression vector pET30a/EsigGOBP1 (Figure S4) that was verified by double enzyme digestion and sequencing; then the correct vector was transferred into the BL21 (DE3) strain, and was induced by IPTG to express recombinant protein. *EsigGOBP1* primers containing the restriction enzyme sites was used, with *EcoR*I in the forward primer (5′-**GAATTC**AGCAGCGCTATATCATCGG-3′) and *Hind*III in the reverse primer (5′-**AAGCTT**TCACAGG CCGTTGTTGCCG-3′). PCR, positive cloned and sequenced were same as 4.2. Plasmids were extracted and digested with *EcoR*I and *Hind*III, and the correct fragment encoding *EsigGOBP1* sequence was purified and sub-cloned into the bacterial expression vector pET30a (+) (Novagen, Madison, WI, USA), and then sequenced for verification. Plasmids containing the correct insert (pET30a- EsigGOBP1) were then transformed into *E. coli* BL21 (DE3) pLysS cells. Isopropyl-β-D-thiogalactopyranoside (IPTG) in 1 mM at 37 °C for 8 h used to induced the expression of *EsigGOBP1*. Samples were sonicated and centrifuged with 4000 rpm at 4 °C for 15 min, then the supernatant and pellet were analyzed by 15% sodium dodecyl sulfate polyacrylamide-gel electrophoresis (SDS-PAGE). EsigGOBP1 appeared as inclusion bodies in pellet, which were purified by Ni-ion affinity chromatography (Qiagen, Hilden, Germany). Soluble protein was obtained by denaturation of the inclusion bodies, elution with 10 M to200 M imidazole rinse buffer, containing 8 M urea in 20 M Tris-Hcl buffer (pH 8.0); followed by renaturation with 0.5 M to 6 M urea renaturation buffer. The protein was concentrated using Amicon Ultra concentrators (Millipore, Billerica, MA, USA) with a 3 kDa cutoff, and purity and concentration were measured by 15% SDS-PAGE and Bradford method respectively.

### 4.5. Fluorescence Binding Assays

N-phenyl-1-naphthylamine (1-NPN) was selected for use as a selectively fluorescent probe to measure the affinity of ligand binding to recombinant *EsigGOBP1* [20,42]. Twelve volatiles (Table 1) from eucalyptus trunks and humus soil under eucalyptus plantation were collected, identified and screened using the dynamic headspace adsorption method, gas chromatograph coupled to mass selective detector, gas chromatograph electroantennal detection and Y-tube olfactometer bioassay [35,36] (Table S1). A fluorescence binding assay was conducted on a multiscan Spectrum Molecular Device SpectraMax i3 (Thermo Scientific, Wilmington, DE, USA) with an excitation wavelength of 337 nm and recording of emission spectra between 380 nm and 520 nm (10 nm slit widths for both excitation and emission). A 2 μM solution of EsigGOBP1 was prepared in 20 mM Tris-HCl buffer (pH 7.4), and the ligands were dissolved in chromatographically pure methanol as 1 mM stock solutions. The affinity of EsigGOBP1 for the labeled probe was determined by adding aliquots of the 1-NPN stock solution to give final concentrations of 2–24 μM. The affinity of EsigGOBP1 for the different ligands was evaluated by competitive binding assays with both 1-NPN and EsigGOBP1 at 2 μM, and final concentrations of twelve ligands in the range of 2–24 μM with six repetitions. Intensity values corresponding to maximum fluorescence emission were plotted against free ligand concentrations to determine dissociation constants. *K_1-NPN_* values were calculated by Scatchard plots to linearize curves. Dissociation constants of the competitors (*K_i_*) were calculated from the corresponding IC_50_ values by the following equation: *K_i_* = [IC_50_]/(1 + [1-NPN]/*K_1-NPN_*) [67].

### 4.6. Molecular Docking and Simulation of the Molecular Dynamics of the EsigGOBP1-Alpha-Phellandrene Complex 

Molecular docking was conducted to investigate the mode of ligand binding. Autodock Tool 1.5.6 was used to perform docking calculations for EsigGOBP1 and volatile selected from FCBA [68]. The information of all ligands was searched in PubChem (https://pubchem.ncbi.nlm.nih.gov/; accessed on 21 June 2022) (Table S1). Evaluation and sorting were performed based on the complex conformation, free binding energy and other parameters. Conformations with reasonable binding sites and the lowest free binding energies were selected. A 50 ns molecular dynamics (MD) simulation with a 2-fs time step was performed to analyze conformational changes of the EsigGOBP1-alpha-phellandrene complex, which was selected based on their high binding affinity to EsigGOBP1 in FCBA and binding energy. The initial conformation for the simulation was obtained by molecular docking. The production runs were accomplished with the GROMACS package (version 2019.6) using the amber99sb-ildn force field for protein and the tip3p water model [69]. Alpha-phellandrene was parametrized using the AMBER force field (GAFF) [70]. The system energy was minimized with the conjugate gradient method, and the whole system was balanced with a V-rescale thermostat and 1 bar with the Parrinello-Rahman barostat. During the 50 ns simulation, the long-range electrostatic interactions was treated using the particle mesh Ewald (PME) method. The covalent bonds of hydrogen atoms were constrained with the Linear Constraint Solver (LINCS) algorithm. Meanwhile, the cutoff radius for non-bonded interactions was set to 10 Å in all stages of the simulation. The root-mean-square deviation (RMSD) was obtained as a measure of the stability of the EsigGOBP1-alpha-phellandrene complex, while the root-mean-square fluctuation (RMSF) was used to determine the flexibility of a region of *EsigGOBP1*. Dominant conformations were obtained to observe the interaction between *EsigGOBP1* and alpha-phellandrene by cluster analysis, and visualized with the Discovery Studio 2019 (DS, Accelrys Inc., San Diego, CA, USA).

## 5. Conclusions

*EsigGOBP1* showed the only and highest expression in the heads of 3rd instar larvae of *E. signifer*, indicating that it was the key OBP in *E. signifer* larvae. Results on the dynamic expression of *EsigGOBP1* to volatile exposures, FCBA and molecular docking analyses, together showed that it had a high binding affinity to alpha-phellandrene in 3rd instar larvae of *E. signifer*. We also identified MET49 and GLU38 as the key binding sites in *EsigGOBP1*. These results demonstrated that *EsigGOBP1* is a key olfactory protein, which binds to the main volatile from eucalyptus trunk, alpha-phellandrene; they also suggest that this odorant-binding protein involved in host selection of *E. signifer* larvae, can serve as a molecular target for the control of this pest; *however*, further studies on the binding and releasing mechanisms underlying this *EsigGOBP1-*alpha-phellandrene complex, and the downstream interactions among odorant receptors are needed. 

## Figures and Tables

**Table 1:** Binding of different compounds to recombinant GOBP1 of *E. signifer*.

EsigGOBP1			
Ligands	IC_50_ (µM)	K_i_ (µM)	Binding Energy (kcal/mol)
Camphene	7.870	0.988	−5.21
Eucalyptol	10.130	1.272	−4.26
Benzene, 1,2-diethyl-	6.305	0.792	−4.11
alpha-Pinene	9.860	1.238	−4.54
β-Pinene	10.520	1.321	−4.55
alpha-Phellandrene	7.080	0.889	−5.00
n-Butyl ether	9.400	1.180	−3.56
D-limonene	11.410	1.433	−4.79
Butyl acrylate	7.180	0.901	−3.57
1,3,5-trimethyl-benzen	36.658	4.602	−4.12
Naphthalene	21.454	2.694	−4.57
2-Phenyl-2-propanol	22.024	2.765	−3.98

## Data Availability

Not applicable.

## Supplementary Materials

The supporting information can be downloaded at: https://www.mdpi.com/article/10.3390/ijms23169269/s1.
