# Supplementary material for: EsigGOBP1: The Key Protein Binding Alpha-Phellandrene in Endoclita signifer Larvae
Source: Int J Mol Sci. 2022 Aug 17;23(16):9269. doi: 10.3390/ijms23169269 (PMC9409361; doi:10.3390/ijms23169269)
Supplement: Supplementary file 1 [file ijms-23-09269-s001.zip › ijms-1852406-supplementary.pdf]

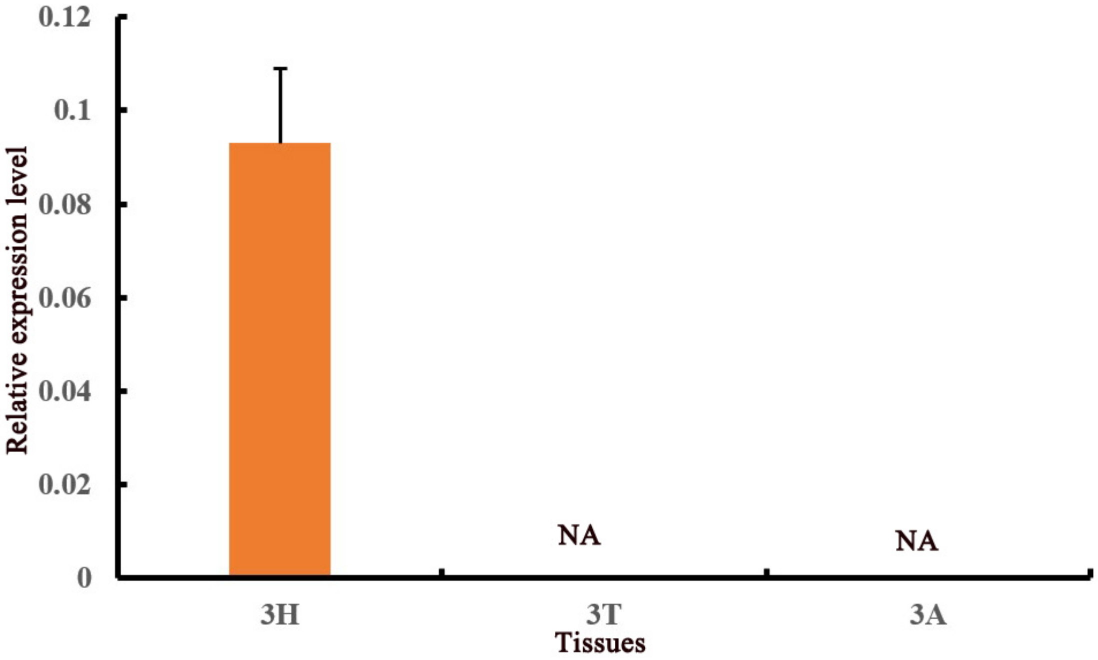

Figure S1. Expression profile of EsigGOBP1 in tissues of 3rd *E. signifer* larvae

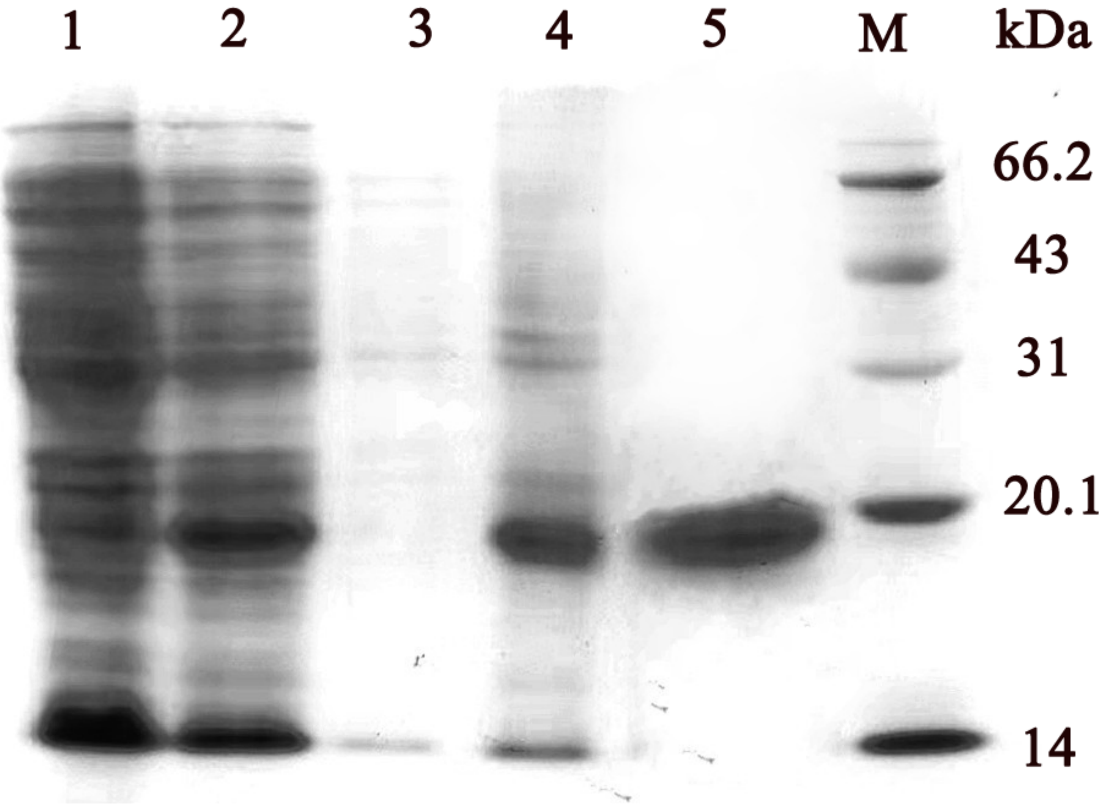

Figure S2. Analysis of the expression of EsigGOBP1 protein by SDS-PAGE.

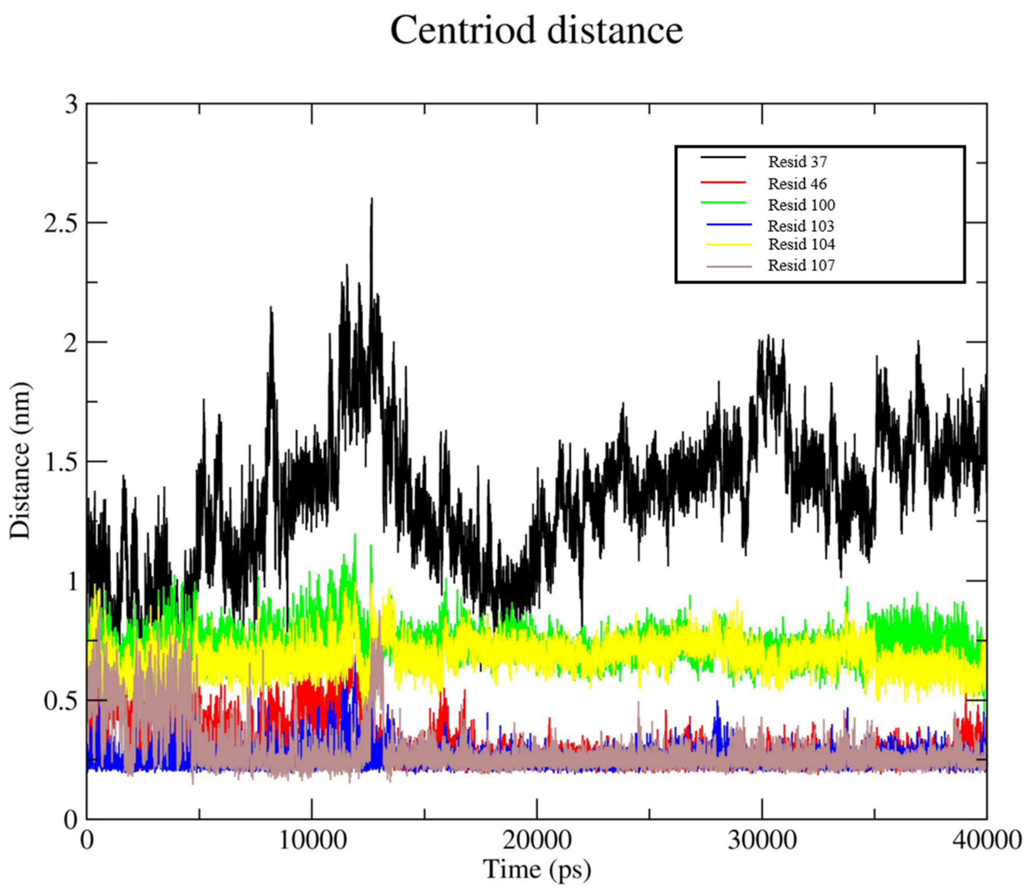

Figure S3. Centroid distance of six key amino acid residue.

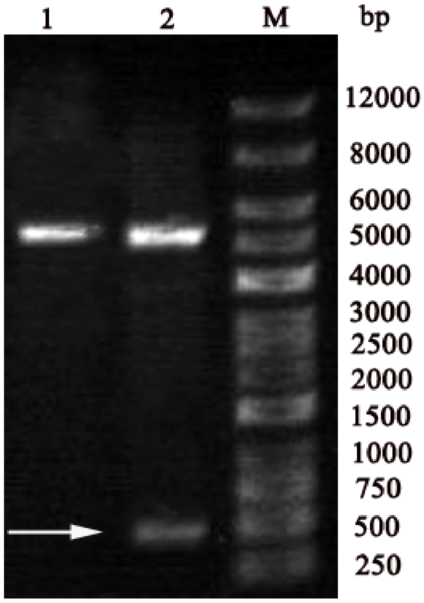

Figure S4. Double enzyme product of EsigGOBP1 and cloning vector pET30.

Table S1. Chemical compounds information.

Compounds	Purity	Company	3rd larvae	5th larvae	Molecular docking
Eucalyptol	99%	Macklin	*	*	*
o-Cymene	98%	Aladdin	*	*	—
Camphene	96%	Macklin	*	*	*
4-Ethylacetophenone	97%	Aladdin	*	*	—
Benzene, 1,2-diethyl-	97%	Macklin	—	*	*
Benzene, 1-ethyl-2-methyl-	99%	Rhawn	—	*	—
n-Butyl ether	99%	Rhawn	*	*	*
alpha-Pinene	99%	Macklin	—	*	*
β-Pinene	98%	Macklin	—	*	*
.alpha.-Phellandrene	99%	Macklin	*	*	*
Naphthalene	98%	Aladdin	*	—	*
3-Carene	90%	Klamar	*	—	—
D-limonene	99%	Macklin	—	—	*
2-Phenyl-2-propanol	97%	Macklin	—	—	*
1, 3,5-trimethyl-benzen	99%	Macklin	—	—	*
Butyl acrylate	99%	Macklin	—	—	*

* means have done the experiment; — means not done the experiment.

Table S2. Clusters analysis of EsigGOBP1–alpha-phellandrene complex based on the trajectory of molecular dynamics simulations

System	Clusters	Occurrence [%]
EsigGOBP1–alpha-phellandrene	cluster 1	58.10%
	cluster 2	24.00%
	cluster 3	7.00%
	cluster 4	6.00%
	cluster 5	2.31%
	cluster 6	1.90%
	cluster 7	0.30%
	cluster 8	0.19%
	cluster 9	0.09%
